# Atractylenolide III Attenuates Muscle Wasting in Chronic Kidney Disease via the Oxidative Stress-Mediated PI3K/AKT/mTOR Pathway

**DOI:** 10.1155/2019/1875471

**Published:** 2019-04-18

**Authors:** Mingqing Wang, Rong Hu, Yanjing Wang, Lingyu Liu, Haiyan You, Jiaxing Zhang, Xiaohui Wu, Tingting Pei, Fujing Wang, Lu Lu, Wei Xiao, Lianbo Wei

**Affiliations:** ^1^Shenzhen Hospital, Southern Medical University, Shenzhen 518100, China; ^2^School of Traditional Chinese Medicine, Southern Medical University, Guangzhou 510515, China; ^3^Zhujiang Hospital of Southern Medical University, Guangzhou 510280, China; ^4^The First Affiliated Hospital, Guangzhou University of Chinese Medicine, Guangzhou 510407, China

## Abstract

Oxidative stress contributes to muscle wasting in advanced chronic kidney disease (CKD) patients. Atractylenolide III (ATL-III), the major active constituent of Atractylodes rhizome, has been previously reported to function as an antioxidant. This study is aimed at investigating whether ATL-III has protective effects against CKD-induced muscle wasting by alleviating oxidative stress. The results showed that the levels of serum creatinine (SCr), blood urea nitrogen (BUN), and urinary protein significantly decreased in the ATL-III treatment group compared with the 5/6 nephrectomy (5/6 Nx) model group but were higher than those in the sham operation group. Skeletal muscle weight was increased, while inflammation was alleviated in the ATL-III administration group compared with the 5/6 Nx model group. ATL-III-treated rats also showed reduced dilation of the mitochondria, increased CAT, GSH-Px, and SOD activity, and decreased levels of MDA both in skeletal muscles and serum compared with 5/6 Nx model rats, suggesting that ATL-III alleviated mitochondrial damage and increased the activity of antioxidant enzymes, thus reducing the production of ROS. Furthermore, accumulated autophagosomes (APs) and autolysosomes (ALs) were reduced in the gastrocnemius (Gastroc) muscles of ATL-III-treated rats under transmission electron microscopy (TEM) together with the downregulation of LC3-II and upregulation of p62 according to Western blotting. This evidence indicated that ATL-III improved skeletal muscle atrophy and alleviated oxidative stress and autophagy in CKD rats. Furthermore, ATL-III could also increase the protein levels of p-PI3K, p-AKT, and p-mTOR in skeletal muscles in CKD rats. To further reveal the relevant mechanism, the oxidative stress-mediated PI3K/AKT/mTOR pathway was assessed, which showed that a reduced expression of p-PI3K, p-AKT, and p-mTOR in C2C12 myoblast atrophy induced by TNF-*α* could be upregulated by ATL-III; however, after the overexpression of Nox2 to increase ROS production, the attenuated effect was reversed. Our findings indicated that ATL-III is a potentially protective drug against muscle wasting via activation of the oxidative stress-mediated PI3K/AKT/mTOR pathway.

## 1. Introduction

Protein-energy wasting (PEW), which essentially refers to decreased body protein mass and energy fuels, is a common complication in advanced chronic kidney disease (CKD) and end-stage kidney disease (ESKD) patients, leading ultimately to loss of muscle [1, 2]. Reduced muscle mass, which is closely related to increased mortality risk and reduced quality of life, seems to be the most reliable criterion for the presence of PEW [3]. In addition to the principal mechanisms that mainly participate in muscle wasting in CKD, including activation of the ubiquitin-proteasome system (UPS), caspase-3, and myostatin [4], mounting evidence has suggested that oxidative stress and autophagy are also widely present and strongly associated with muscle wasting in CKD patients [5–7].

Atractylenolide III (ATL-III) is a major active constituent of Atractylodes rhizome. Its reported pharmacological properties include anti-inflammatory, gastroprotective, and neuroprotective effects [8, 9]. Furthermore, treatment of peritoneal macrophages with ATL-III decreased LPS-induced iNOS protein synthesis, suggesting that ATL-III possibly inhibits NO production through downregulating LPS-induced iNOS expression [10]. Thus, ATL-III has been demonstrated to suppress NO production and exerts antioxidation function.

Recent studies have shown that ROS can initiate autophagosome formation and autophagic degradation by acting as cellular signaling molecules [11]. Autophagy, in contrast, serves to reduce oxidative damage and ROS levels through the removal of protein aggregates and damaged organelles such as mitochondria [12]. Gouzi et al. recently identified that the oxidative stress level contributes to the regulation of autophagy in chronic obstructive pulmonary disease (COPD) muscle cells and that autophagy may be involved in the atrophy of COPD skeletal muscles. They observed a significantly increased autophagosome formation and increased ROS concentration in COPD myoblasts, and treatment of the COPD myotubes with the autophagy inhibitor 3-MA could increase their diameter to a level similar to the diameter of healthy myotubes. Interestingly, the antioxidant treatment induced a significant decrease in the LC3-II/LC3-I ratio and the SQSTM1 and BNIP3 expression levels, considered to be two molecular adapters targeting mitochondria to autophagosomes for mitophagy [13]. In addition, the previous studies have shown that ROS production activates FoxO3 and autophagic signaling. The action of FoxO3 is partly inhibited by PGC1a, a factor that is induced by oxidative stressors and that represents an important regulator of intracellular ROS levels [14]. These studies provided evidence of the involvement of oxidative stress in skeletal muscle autophagy.

We hypothesized that ATL-III could reduce skeletal muscle wasting through antioxidant effects. To test this hypothesis, rats with CKD were compared by intragastric administration of ATL-III and normal saline. Serum creatinine (SCr), blood urea nitrogen (BUN), urinary protein, inflammatory factors, and muscle weight were used as indicators of renal function and skeletal muscle atrophy, respectively. Mitochondrial morphology, the activity of superoxide dismutase (SOD), glutathione peroxidase (GSH-Px), and catalase (CAT), and the level of malondialdehyde (MDA) were used to reflect the state of oxidative stress, while autophagosomes (APs), autolysosomes (ALs), and the expression of p62 and LC3-II were studied to explore the effect of ATL-III on autophagy in skeletal muscle tissue in CKD rats. To investigate the underlying mechanism by which ATL-III alleviated oxidative stress and autophagy, the expression levels of p-PI3K, p-AKT, and p-mTOR were detected in C2C12 myoblasts and the skeletal muscles in CKD rats. We also overexpressed Nox2 in C2C12 myoblasts to promote the ROS production, as Nox2 acts as the main source of ROS in skeletal muscle, to verify whether the activation of the PI3K/AKT/mTOR pathway by ATL-III was mediated by oxidative stress.

## 2. Materials and Methods

### 2.1. Animals and Experimental Design

All animal procedures were approved by the Institutional Animal Care and Use Committee (IACUC) of Southern Medical University with the certification number SCXK (Yue) 2016-0041 and usage license number SYXK (Yue) 2016-0167. Male Sprague-Dawley rats at the age of 6-7 weeks (200-250 g) were purchased from the Experimental Animal Center of Southern Medical University, Guangzhou, China, and were maintained in cages at 22°C under a 12 h light/12 h dark cycle with free access to water. The general overall appearance and emotional states of the rats were monitored. The rats were randomly divided into sham operation (*n* = 10) and 5/6 nephrectomy (5/6 Nx) groups. A sham operation was performed on the sham-operated rats, while rats in the 5/6 Nx group underwent surgical resection of the upper and lower thirds of the left kidney and right nephrectomy after one week [15]. After surgery, until the animals were able to ambulate, feed, and drink unaided, they were placed in their home cages. 5/6 Nx rats were randomly divided into two groups: the 5/6 Nx model group (*n* = 10), which was treated with saline water irrigation of the stomach, and the ATL-III-treated group (*n* = 10), which was administered ATL-III at a dose of 2.4 mg/kg every day by gavage. We also added a 3-MA treatment group. 3-MA (Sigma-Aldrich, USA) solution was prepared as follows: 15 mg 3-MA (molecular weight 149.15) powder was dissolved in the 1 ml normal saline by heating solution to 60-70°C immediately before treatment. After successful CKD modeling, the 3-MA treatment group rats (*n* = 10) were injected with 15 mg/ml 3-MA solution at a dose of 15 mg/kg via the intraperitoneal route once every day for 5 weeks. The rats were monitored daily during the first week, and then once every two days until they were euthanized. Through the experience, the general overall appearance of the rats was evaluated. The monitoring criteria included (a) pain, monitored by body posture and movement; (b) wound inflammation, judged by cracking and swelling; (c) wound bleeding; (d) mental states, diagnosed by abnormal gait, hemiplegia, or coma; and (e) body weight.

When the rats were sacrificed after oral administration of ATL-III for 5 weeks, the gastrocnemius, soleus and tibial anterior (TA) muscles, and serum were collected. The gastrocnemius and tibial anterior muscles for the dry weight measurement were dissected from the carcass and dried to constant weight in the drying oven. The process of dosage selection is as follows: the recommended dose of atractylenolide is 15 g/d for an adult (60 kg) according to clinical application. The content of atractylenolide III in atractylenolide was 1.552 mg/g extract by high-performance liquid chromatography, so the dose of ATL-III should be 23.28 mg/60 kg, namely, 0.388 mg/kg for patients. A human equivalent dose of 0.388 × 6.2 (the conversion coefficient) = a rat dose of 2.4 mg/kg [16]. Therefore, we chose doses of 2.4 mg/kg for rats in this study.

### 2.2. Blood and Urine Examination

At the end of the study, the rats were anesthetized by intraperitoneal injection of sodium pentobarbital at the concentration of 40 mg/kg. Five minutes later, the rats were fixed on the rat operating table. After routine disinfection, the abdominal cavity was cut along the midline of the abdomen with surgical scissors. The operator holds the puncture needle on the right side, the needle tip is inclined downward to the abdominal aorta, the needle angle is about 25°-30°, and the depth is about 5 mm. The blood can be sucked up, and 10 ml of blood was collected to vacuum tubes. Before collection of serum, blood was centrifuged at 3,000 rpm for 15 minutes. Aortic blood obtained from anesthetized rats was used to measure SCr, BUN, and serum albumin.

The 24 h urine samples were collected using metabolic cages, and urinary protein excretion was tested with the commercial kit (Tonein-TP II, Tokushima, Japan) according to the instructions of the manufacturer.

### 2.3. Detection of Inflammatory Factors

The levels of inflammatory factors, such as serum CRP (R&D Systems, USA; DY1744), TNF-*α* (SRTA00), IL-1*β* (R&D Systems, USA; SRLB00), and IL-6 (R&D Systems, USA; SR6000B) in serum, were measured by colorimetric enzyme-linked immunosorbent assay (ELISA). And the levels of TNF-*α*, CRP, IL-1*β*, and IL-6 in gastrocnemius muscle in CKD rats were measured by qPCR. The primer sequences used are as follows: GAPDH: sense: 5-CTGGAGAAACCTGCCAAGTATG-3, antisense: 5-GGTGGAAGAATGGGAGTTGCT-3; IL-1*β*: sense: 5-TGATGAAAGACGGCACACCC-3, antisense: 5-TGTCCCGACCATTGCTGTTT-3; TNF-*α*: sense: 5-GCCACCACGCTCTTCTGTCTA-3, antisense: 5-CGCTTGGTGGTTTGCTACGA-3; IL-6: sense: 5-AAGCCAGAGTCATTCAGAGCAA-3, antisense: 5-GTCTTGGTCCTTAGCCACTCCT-3; and CRP: sense: 5-CCTTCGTATTTCCCGGAGTGTC-3, antisense: 5-CTCACATCAGCGTGGGCATAG-3.

Because macrophages play an important role in skeletal muscle repair and remodeling, the markers of M1 proinflammatory macrophages (CD68+/CD86+) and M2 anti-inflammatory macrophages (CD68+/CD206+) in muscle sections of different groups were also detected by performing immunofluorescence.

### 2.4. Transmission Electron Microscopy (TEM)

The fresh gastrocnemius muscle was sectioned in 40 mm^3^ blocks, immersed for 1 h in fixative solution (0.5% glutaraldehyde, 2% paraformaldehyde, 7% saccharose, and 4% polyvinylpyrrolidone in 0.1 M cacodylate buffer), and rinsed with 0.1 M PBS buffer. These muscle blocks were recut into smaller samples of about 10 mm^3^ and fixed with another fixative solution (2% osmic acid in 0.1 M cacodylate buffer) for 1 h. Subsequently, the samples were dehydrated, infiltrated, and embedded in Epon 812 at 60° for 48 h and cut into longitudinal or transversal sections 0.1 *μ*m thick. These sections were stained with 1% uranyl acetate and Reynolds's lead citrate. They were observed on a Leica UC7 HT7700 (HITACHI) microscope to analyze the ultrastructure of mitochondria.

Four images were taken randomly under TEM from each sample; then, the number and ultrastructure of mitochondria, including the number of Aps and ALs, were evaluated with ImageJ. For example, we determined the number, area (*μ*m^2^), and Feret diameter (*μ*m) of the mitochondria, which describe the shape of the mitochondria. Data obtained from the four random images in each sample were averaged, and the results of the individual rats within a certain group were also averaged. These data were used for comparison among three groups.

### 2.5. Hematoxylin-Eosin (H&E) Staining

Fresh gastrocnemius muscle tissues were dissected from the carcass, rinsed with 0.1 M PBS buffer, fixed with 4% paraformaldehyde for 48 h, dehydrated using graded ethanol, and then embedded in paraffin. The samples were sliced into 4 mm paraffin-embedded pieces, dewaxed with xylene, rehydrated in a graded ethanol series to water, and stained with hematoxylin and eosin (H&E). Sections were dehydrated in a graded alcohol series to thoroughly remove the water in the tissues. Then, four images were taken randomly for each sample, and the myofiber cross-sectional area of these images within a certain group was analyzed with ImageJ.

### 2.6. Cell Culture and Treatments

The skeletal muscle cell line C2C12 myoblasts were cultured with high-glucose DMEM (Gibco, New York, USA) supplemented with 10% fetal bovine serum (FBS) (Gibco, New York, USA), 2 mmol L-glutamine, and 100 U/ml penicillin-streptomycin in a humidified atmosphere containing 5% CO_2_/95% air at 37°C. To detect the effect of ATL-III on cell toxicity, C2C12 myoblasts were treated with different concentrations of ATL-III (0 *μ*M, 20 *μ*M, 30 *μ*M, 50 *μ*M, and 100 *μ*M) for 24 h, and then, the viability of cells was detected using MTT assay. In order to find the optimal concentration of ATL-III in vitro assay, TNF-*α* (#061454-1, PeproTech, USA) was used to induce oxidative stress and inflammation models in C2C12 myoblasts [17–19], and the C2C12 myoblasts were cultured with incubation of different concentrations of ATL-III (20, 30, and 50 *μ*M) in the presence or absence of TNF-*α* (20 ng/ml) for 120 h (cell proliferation assay) or 24 h (cell apoptosis assay); then, the cell proliferation and apoptosis were detected. To explore the effect of ATL-III on oxidative stress and autophagy, C2C12 myoblasts were divided into three groups: a control group cultured with only DMEM with 10% FBS, a TNF-*α* induction group treated with 20 ng/ml TNF-*α* for 24 h, and an ATL-III-treated group incubated with optimal concentration of ATL-III in the presence of TNF-*α* (20 ng/ml) for 24 h. Then, the cell autophagy and oxidative stress were detected.

### 2.7. MTT Assay

C2C12 cells were seeded in 96-well plates (4,000 cells/well), cultured at 37°C with 5% CO_2_ for 24 h, and then treated as mentioned previously. Subsequently, the medium was removed and replaced by 100 *μ*l of MTT solution (0.5 mg/ml) in DMEM medium. After incubation at 37°C in the humid environment with 5% CO_2_ for 4 h, the MTT containing medium was gently removed and the purple formazan crystals were dissolved in 100 *μ*l dimethyl sulfoxide (DMSO). The plate was shaken in the dark for 15 min, and then, the absorbance at 490 nm was measured using a microplate reader (S5 Versa Analyzer, USA). The proliferation curves of different groups were plotted and compared with each other. All the results were averaged from three replications.

### 2.8. Cell Apoptosis Assay

Hoechst 33342 (C0030, Solarbio, China) is a type of fluorescent dye that can be used to evaluate cell apoptosis. C2C12 myoblast cells were seeded in 6-well plates. After 24 h of culture, the cells were treated with ATL-III (0 *μ*M, 20 *μ*M, 30 *μ*M, and 50 *μ*M) in the presence or absence of TNF-*α* (20 ng/ml) for 24 h. Then, the supernatant was removed, and the cells were washed with cold PBS three times and incubated with Hoechst 33342 dye (10 *μ*g/ml) in the dark for 20 min at 37°C. After rinsing with cold PBS, the cells were immediately observed with a fluorescence microscope. Cells with condensed/fragmented nuclei and high intensity of blue staining were identified as apoptotic cells, which were calculated from five random microscopic fields of view in different groups. The percentage of apoptotic cells was quantified as apoptotic cells relative to the overall number of cells observed.

### 2.9. Immunofluorescence

Immunofluorescence staining was done by following the manufacturer's instructions. The muscle tissue sections were incubated with corresponding primary antibodies at 4°C overnight, followed by incubation with corresponding secondary anti-rabbit or anti-mouse Alexa 488 or 555 from Thermo Fisher Scientific (1 : 1000 in PBS and BSA 2%) for 1 h at room temperature. The primary antibodies for muscles are as follows: CD68 (1 : 100, Abcam, ab31630), CD86 (1 : 100, Santa Cruz, SC-28347), and CD206 (1 : 100, Affinity, DF4149).

For cells, C2C12 myoblasts in different groups were seeded on coverslips overnight. After fixation with 4% formaldehyde for 20 min, the cells were permeabilized with 0.25% Triton X-100 in PBS for 10 min and incubated with a 5 mg/ml bovine serum albumin (BSA) blocking solution for 30 min and LC3A/B primary antibody (1 : 400, AF5402, Affinity, USA) at 4°C overnight. The cells were washed with PBS and incubated with secondary antibody (donkey anti-rabbit IgG-Alexa Fluor 594; Thermo Fisher Scientific, USA) at 37°C for 1 h.

For muscles and cells, the DNA was stained with 4′,6-diamidino-2-phenylindole (DAPI) (0.1 *μ*g/ml) in PBS for 5 min. Then, five immunofluorescence images were taken randomly for one sample within a certain group with a fluorescence microscope (Eclipse Ti, Nikon, Japan), and the fluorescence intensity of LC3 in different groups was analyzed by ImageJ.

### 2.10. ROS Measurements

The intracellular generation of ROS was measured using a 2′,7′-dichlorofluorescein diacetate (H2DCFDA) fluorescent probe (GeneCopoeia, C263, USA). C2C12 myoblasts were seeded in triplicate at a density of 5 × 10^3^ cells per well in 96-well plates. After 24 h of culture, the cells were treated as mentioned previously in Section 2.6. Then, the cells were incubated with DCFH-DA (10 *μ*M) at 37°C for 30 min in the darkness. The fluorescence intensity of the oxidized probe in each well was measured at 485 nm and 530 nm using a fluorescence microplate reader (Synergy HTX Multi-Mode Reader, USA). Results are expressed as the intracellular ROS level relative to the control group. Four representative images in different groups were also taken randomly under a fluorescence microscope.

### 2.11. Analysis of Oxidative Stress Markers

Rat skeletal muscle tissue was quickly removed, homogenized in cold PBS on ice, and preserved at -80°C. Approximately 60 mg of skeletal muscle tissue was obtained by ultrasonic grinding and centrifuged at 12,000 rpm for 15 minutes at 4°C. Before collection of serum, blood was centrifuged at 3,000 rpm for 15 minutes. All supernatants of gastrocnemius and serum were collected for oxidative stress analysis. After digesting the C2C12 cells in different groups with trypsin, the culture medium was centrifuged at 1,000 rpm for 10 minutes at room temperature and the supernatant was discarded to retain the cell precipitation. Then, SOD, GSH-Px, and CAT activity and the MDA level were measured in gastrocnemius tissue, serum, and C2C12 cells using the detection kits (SOD: A00-1; MDA: A003-1; CAT: A007-1; GSH-Px: A005, Jiancheng Bioengineering Ltd., Nanjing, China) in accordance with the manufacturer's instructions.

### 2.12. Overexpression of Nox2 in C2C12 Myoblasts

Nox2 (NM_007807.5) was cloned into a PCDH lentiviral vector with the following primers: sense, 5′-ATGGGGAACTGGGCTGTGAATG-3′; antisense, 5′-TTAGAAGTTTTCCTTGTTGAAAATG-3′. Transient transfection of the Nox2 overexpression plasmid by Lipofectamine 3000 (Invitrogen, USA) was performed according to the manufacturer's instructions.

### 2.13. Western Blotting Analysis

The frozen tissues were ground into powder with an ultrasound after adding radio immunoprecipitation assay (RIPA) lysis buffer containing protease and phosphatase inhibitors. The samples were centrifuged for 5 minutes at 12,000 ×g, and the supernatant was collected for protein quantitation by a bicinchoninic acid (BCA) assay. Equal volumes (50 mg) of protein were separated using SDS-PAGE and transferred to nitrocellulose membranes. The membranes were incubated overnight at 4°C in 5% skim milk with the following primary antibodies: p-PI3K, p-AKT (Ser473), p-mTOR (all 1 : 1000, Cell Signaling Technology, USA), phospho-p62, LC3-II (all 1 : 1000, Affinity, USA), Nox2 (1 : 1000, ab31092, USA), and GAPDH (1 : 1000, Cell Signaling Technology, USA). The membranes were then washed and incubated using a secondary anti-rabbit IgG or anti-mouse IgG (all 1 : 10000, Beyotime Institute of Biotechnology, China). The corresponding secondary antibodies goat anti-mouse IgG (H+L)-HRP (BS12478) and goat anti-rabbit IgG (H+L)-HRP (BS13278) were all purchased from Bioworld Technology Company, and all the dilution for Western blotting is 1 : 5000. Band visualization was performed using an ECL Western Blotting Substrate kit (Millipore, 1622301, USA).

### 2.14. Statistical Analysis

The analysis among the three different groups was performed using one-way ANOVA. All the statistics were performed with SPSS 13.0. Data are expressed as the means ± SD, and a two-sided test with *P* < 0.05 was considered statistically significant.

## 3. Results

### 3.1. ATL-III Improved Renal Function, Inhibited CKD-Induced Muscle Atrophy, and Alleviated Inflammation in CKD Rats

Tables 1 and 2 show renal function and muscle weight in the sham, 5/6 Nx model, and ATL-III treatment groups. The levels of BUN, SCr, serum albumin, and 24 h urinary protein were within normal ranges in the sham group, while they were increased in the CKD group. However, after administration of ATL-III for 5 weeks, significant reductions in the levels of BUN, SCr, and urinary protein were observed in the ATL-III administration group, while the serum albumin level showed an opposite result compared with the model group (*P* < 0.001) (Table 1).

Body weight, muscle weight, and muscle fiber area were measured as direct indicators of muscle atrophy. The body weight and wet weight of the gastrocnemius (Gastroc), soleus (Sol) muscle, and tibial anterior (TA) muscles were significantly decreased (*P* < 0.01) in the model group compared with the sham group, while they were increased in the ATL-III treatment group (*P* < 0.05) (Table 2).

**Figure 1:**
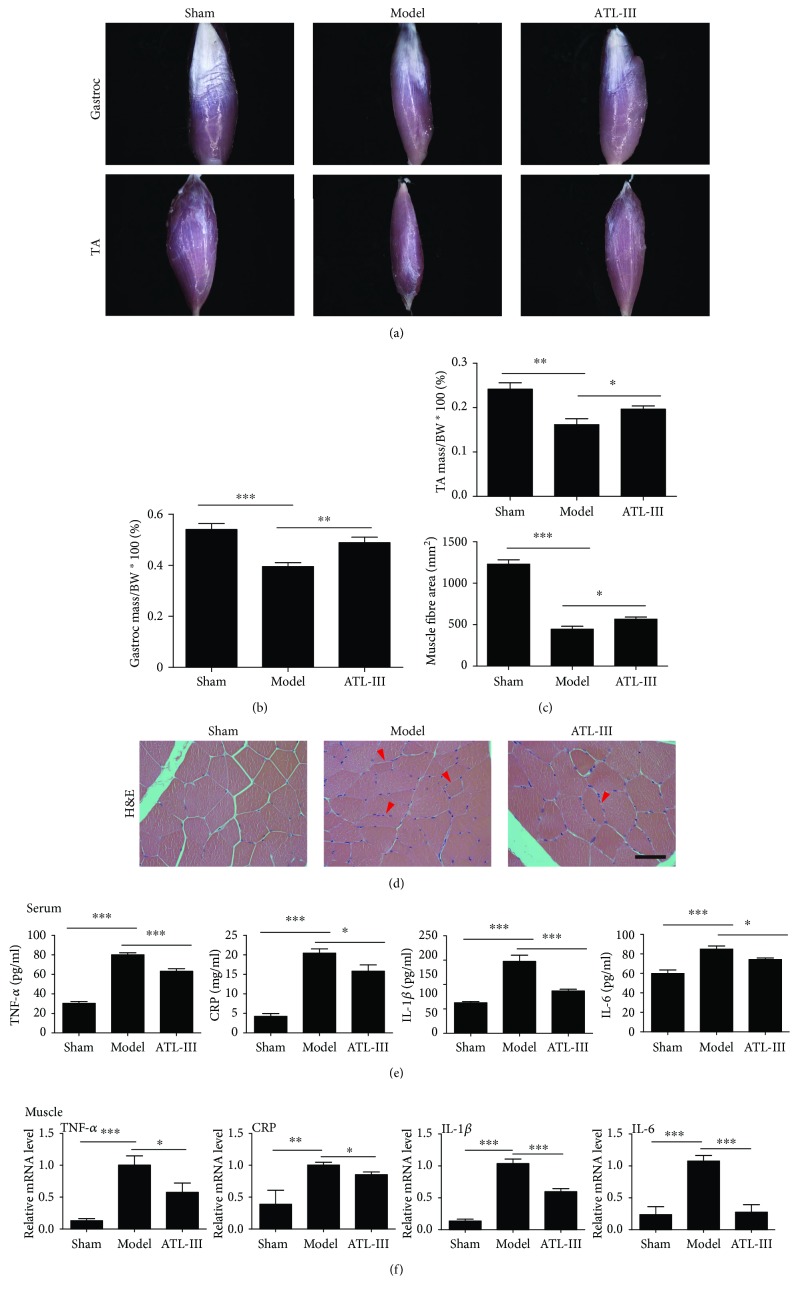
ATL-III prevents CKD-induced muscle atrophy and reduces the levels of inflammatory factors in 5/6 Nx model rats. The animals were sacrificed after treatment for 5 weeks, and the gastrocnemius (Gastroc) and tibial anterior (TA) muscles were observed by a stereoscope to obtain images (*n* = 6/group) (a). The proportion of Gastroc (b) and TA (c) muscle masses normalized to total body weight was quantified and displayed. The volume of transverse muscle fibers was evaluated by H&E staining (d). Representative images of tissue sections from rats in the sham, model, and ATL-III groups are shown by the microscope (400×). The red arrows indicate myofibers affected by atrophy. The average muscle fiber area (*μ*m^2^) of different groups was compared by one-way ANOVA. Inflammatory factors TNF-*α*, CRP, IL-1*β*, and IL-6 in serum and gastrocnemius muscle tissue were detected by ELISA (e) and qPCR analysis (f). Data are presented as the mean ± SD for three independent experiments. ^∗^ *P* < 0.05, ^∗∗^ *P* < 0.01, and ^∗∗∗^ *P* < 0.001.

The gastrocnemius and tibial anterior muscle volumes were larger in the ATL-III treatment group than in the model group, although they were smaller than in the control group (Figure 1(a)). Furthermore, the ratio of gastrocnemius and tibial anterior muscle dry weight normalized to body weight in the model group showed similar trends (Figures 1(b) and 1(c)). The cross-sectional area of the muscle fiber is typically used to describe muscle atrophy, thus avoiding potential confounding factors related to changes in extracellular space. We observed a smaller average cross-sectional area in 5/6 Nx model rats than in the sham rats (*P* < 0.001). Similarly, after ATL-III treatment, the cross-sectional area was increased compared to the model group, though it was much smaller than the sham group (Figure 1(d)).

As inflammation usually exists in patients with CKD, which in turn contributes to the progression of CKD, we also detected the levels of various cytokines such as TNF-*α*, CRP, IL-1*β*, and IL-6 both in serum and gastrocnemius muscle of CKD rats by ELISA and qPCR. In the model group, all the proinflammatory cytokines were remarkably increased compared with those in the sham group (*P* < 0.01). In contrast, the level of TNF-*α*, CRP, IL-1*β*, and IL-6 was sharply reduced after treatment with ATL-III for 5 weeks (*P* < 0.05) (Figures 1(e) and 1(f)).

Besides, to detect whether the inflammation could affect the number of total macrophages and whether ATL-III treatment affect the transition from M1 to M2 phenotype in macrophages in CKD muscles, CD68+ (the marker of total macrophages), CD86+ (the marker of M1 macrophages), and CD206+ (the marker of M2 macrophages) macrophages in gastrocnemius muscle sections of the three groups were detected by performing immunofluorescence. The result showed that the number of total macrophages (CD68+) significantly increased in the model group compared to the sham group; after ATL-III treatment, the number of CD68+ macrophages slightly decreased, but there was no significant difference between the model group and the ATL-III treatment group (Figures S1a, S1b, and S1c). The number of M1 macrophages (CD68+/CD86+) increased significantly in the CKD model group compared to the sham group (*P* < 0.001), while decreased after ATL-III treatment (*P* < 0.001) (Figures S1a and S1d). As for M2 macrophages (CD68+/CD206+), the number in both the sham and model groups was very small, and also there was no significant difference between the two groups, but after ATL-III treatment, the number of M2 macrophages (CD68+/CD206+) increased significantly (*P* < 0.01) (Figures S1b and S1e). The result indicated that the inflammation state in CKD muscle increased the number of total macrophages, and ATL-III might promote the transition from M1 to M2 phenotype in macrophages in gastrocnemius muscle tissue.

Taken together, these results indicated that ATL-III improved kidney function and alleviated microinflammation in 5/6 Nx-induced CKD model rats. The treatment of ATL-III in CKD rats can also promote the transformation of M1 to M2 in gastrocnemius muscle tissue. Loss and damage of skeletal muscle during CKD were also improved by ATL-III.

### 3.2. ATL-III Extenuated Mitochondrial Swelling and Increased the Activity of Antioxidant Enzymes (SOD, GSH-Px, and GST) in CKD Model Rats

**Figure 2:**
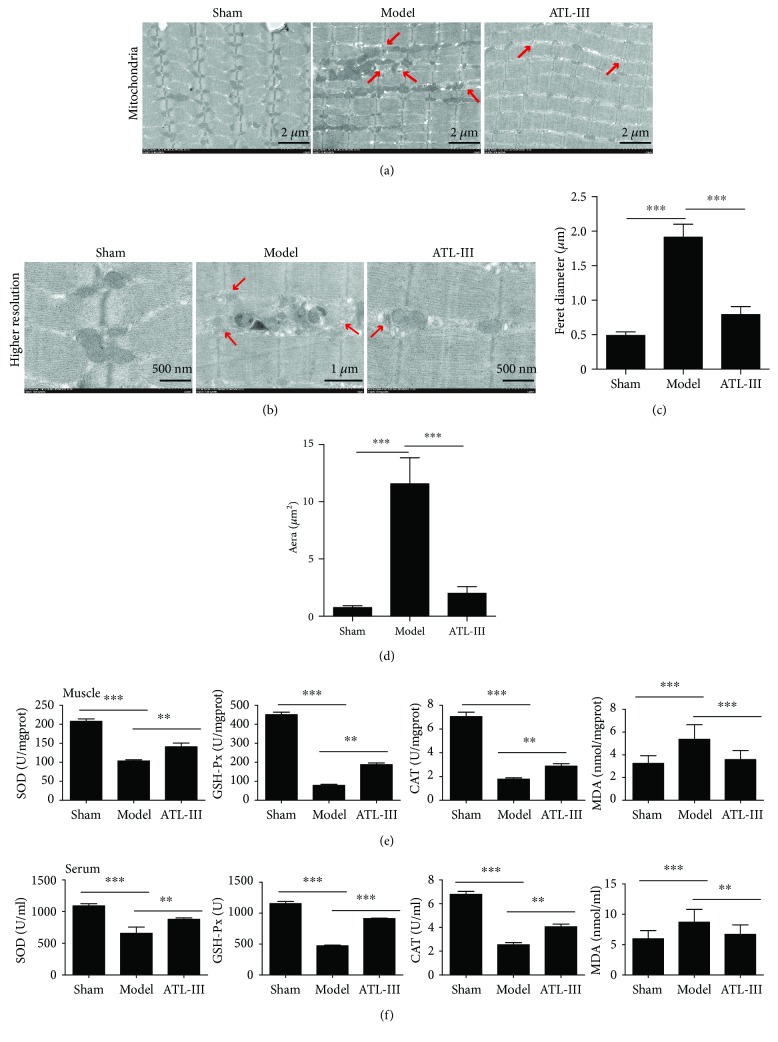
Altered mitochondrial morphology and peroxidase activity in gastrocnemius muscle tissue and serum in CKD rats. Rat gastrocnemius tissue obtained from the sham, model, and ATL-III groups was subjected to transmission electron microscopy (TEM) analysis, and the mitochondrial structure was compared. Representative electron micrographs showed changes in mitochondrial morphology of CKD. Mitochondria were swollen and showed a disordered arrangement and membrane ruptures or large vacuoles. The red arrows represent typical swollen mitochondria (a). Higher magnification views of the corresponding area are shown in (b). The mitochondrial average Feret diameter (c) and area (d) in gastrocnemius muscles from different groups were calculated and displayed on the right side. The activity of SOD, MDA, and GSH-Px and the level of CAT in muscle (e) and serum (f) are shown in the histograms. ^∗∗^ *P* < 0.01 and ^∗∗∗^ *P* < 0.001. SOD: superoxide dismutase; MDA: malondialdehyde; GSH-Px: glutathione peroxidase; CAT: catalase.

Next, we examined the influence of ATL-III on oxidative stress. First, the mitochondrial morphology of gastrocnemius muscle was severely damaged, and also the vacuolization, mitochondrial cristae, membrane loss, and the accumulation of enlarged or highly interconnected mitochondria in skeletal muscle were observed in 5/6 Nx model rats. However, the destroyed structures were restored to the morphology observed in the sham group after ATL-III administration, as the mitochondrial crista arrangement tended to be dense and with reduced swelling (Figures 2(a) and 2(b)). Furthermore, ATL-III also reduced the Feret diameter (*μ*m) and enlarged area (*μ*m^2^) of the mitochondria induced by CKD in gastrocnemius muscle (Figures 2(c) and 2(d)).

Biomarkers of the antioxidant defense system and lipid peroxidation, namely, catalase (CAT), superoxide dismutase (SOD), glutathione peroxidase (GSH-Px), and malondialdehyde (MDA), were used as indicators of oxidative damage in serum and tissues. Compared with sham-operated rats, the levels of antioxidant enzymes in the gastrocnemius muscle and serum of 5/6 Nx model rats significantly decreased, but partially increased after ATL-III treatment. We found that the antioxidant defense system in gastrocnemius muscles was impaired, while ROS activity was increased, as indicated by the lower levels of SOD, CAT, and GSH-Px in CKD rats. Correspondingly, elevated levels of MDA in 5/6 Nx model rats both in tissues and serum appeared to follow a downward trend after treatment with ATL-III for 5 weeks (*P* < 0.01) (Figures 2(e) and 2(f)).

### 3.3. ATL-III Suppressed Autophagy in Skeletal Muscle in CKD Rats

**Figure 3:**
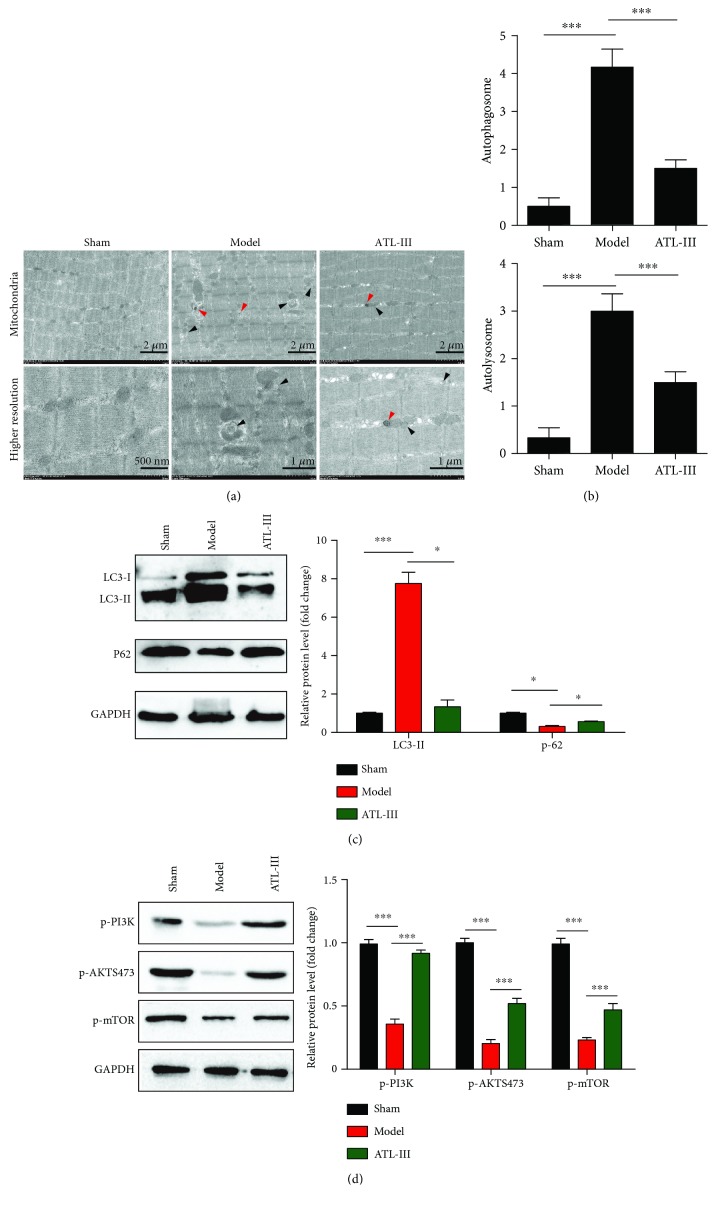
Typical autophagosomes and autolysosomes of gastrocnemius slices under TEM. The skeletal muscle of rats was obtained after 5 weeks of gavage. Autophagosomes and autolysosomes of gastrocnemius were observed by TEM. Representative TEM images showing autophagosome structures (denoted by black triangles) and autolysosome structures (denoted by red triangles) (a). The number of autophagosomes and autolysosomes was significantly higher in the model group than in the sham group. However, after ATL-III treatment, the numbers of both autophagosomes and autolysosomes were decreased (b). Western blotting analysis showed that the LC3-II expression was significantly higher in the model group than in the sham group (*P* < 0.001) but was decreased in the ATL-III-treated group (*P* < 0.05); the expression of P62 followed an opposite trend (c). The expression of p-PI3K, p-AKT (Ser473), and p-mTOR in muscle lysates of different groups by Western blots. The results showed that the expression levels of these three proteins decreased significantly in CKD rats compared to the sham group but could be restored by ATL-III treatment (d). ^∗^ *P* < 0.05 and ^∗∗∗^ *P* < 0.001.

Many studies have proven that increased autophagy can be induced by ROS. To investigate the effect of ATL-III on autophagy in skeletal muscle atrophy, we detected the formation of autophagosomes (APs) and autolysosomes (ALs) under TEM in rat skeletal muscle tissue. Autophagosomes are the key structure in macroautophagy and are characterized by a spherical structure with double-layer membranes, while autolysosomes include the fusion of autophagosomes and lysosomes (Figure 3(a)). The results showed distinctly more APs and ALs in the model group than in the control group (*P* < 0.001), but after treatment with ATL-III, both the numbers of APs and ALs were significantly decreased (*P* < 0.001) (Figure 3(b)). To further verify the inhibitory effect of ATL-III on autophagy, we added an autophagy inhibitor (3-MA) treatment group for CKD rats to confirm whether similar results could be achieved with ATL-III.

The results showed that 3-MA administration could significantly increase the cross-sectional area of skeletal muscle (Figure S2a) and the weight of gastrocnemius and anterior tibial muscles compared with the model group (Figure S2b) (*P* < 0.01), but its effect was not significant as ATL-III. 3-MA treatment can also reduce the number of autophagosomes (APs) and autolysosomes (ALs) in muscles in CKD rats compared with the model group, suggesting that 3-MA could also improve skeletal muscle atrophy by reducing autophagy (Figure S2c). To obtain further evidence, the levels of LC3 and p62 were measured by Western blotting. Compared with the sham group, we observed that the LC3II expression was significantly increased (*P* < 0.001), while the p62 expression was decreased in 5/6 Nx-induced CKD model rats (*P* < 0.05). However, the upregulation of LC3II and the downregulation of p62 were reversed after ATL-III treatment (*P* < 0.05) (Figure 3(c)). Consequently, these results indicated that ATL-III alleviated the autophagy induced by CKD in skeletal muscles.

To further verify the relevant mechanisms, we detected the expression of p-PI3K, p-AKT (Ser473), and p-mTOR in muscle lysates of different groups by Western blots. The results showed that the expression levels of these three proteins decreased significantly in CKD rats compared to the sham group, but could be restored by ATL-III treatment (Figure 3(d)).

### 3.4. ATL-III Inhibited Apoptosis and Autophagy in C2C12 Myoblasts

**Figure 4:**
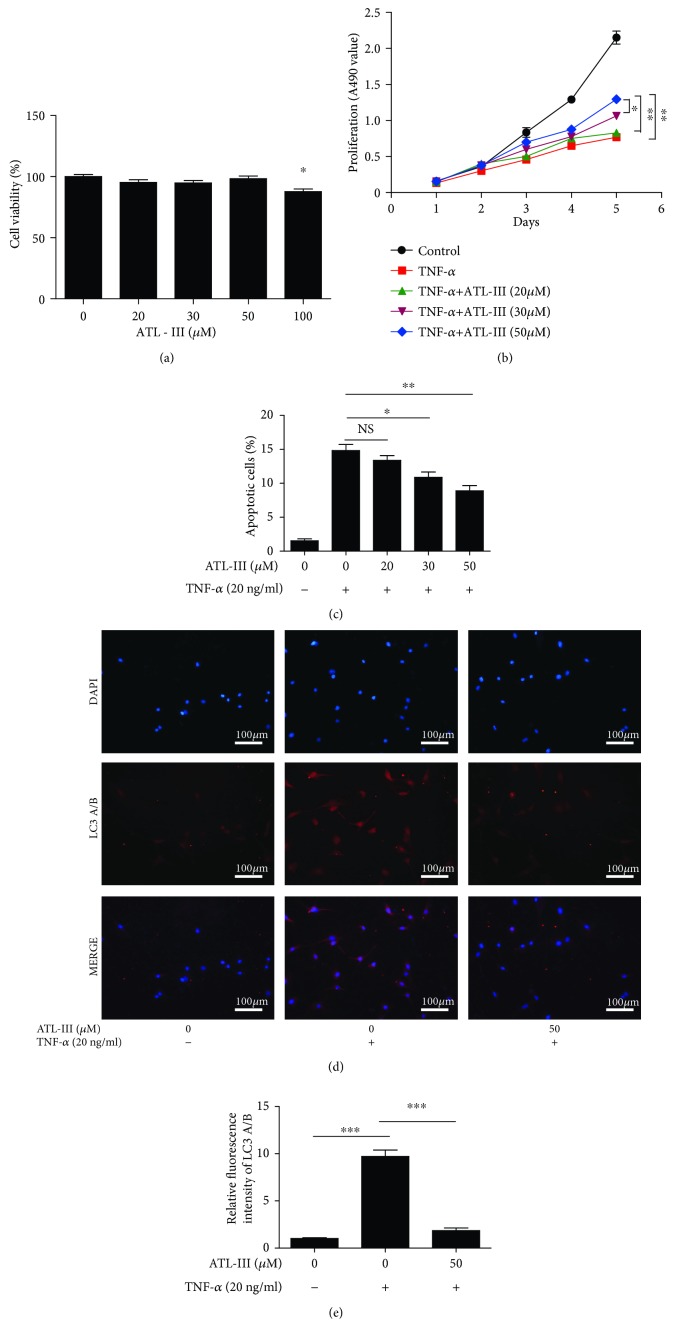
Effects of ATL-III on apoptosis and autophagy in vitro. The effect of different concentrations of ATL-III on the viability of C2C12 myoblasts was tested by MTT, which indicated that 50 *μ*M was the optimal concentration of the drug. Toxicity test of ATL-III showed that low concentration (20, 30, and 50) has no cytotoxicity to C2C12 myoblasts. The values are presented as the means ± SD of five independent experiments. ^∗^ *P* < 0.05 (a). The cell proliferation assay showed that ATL-III at 50 *μ*M displayed the best effect in proproliferation than 20 and 30 *μ*M for C2C12 myoblasts incubated with TNF-*α* (20 ng/ml) (b). ATL-III displayed its antiapoptotic effect in C2C12 myoblasts in the presence or absence of TNF-*α* (20 ng/ml) in a dose-dependent manner (c). The expression of LC3 (d) by immunofluorescence (200×) in different groups was displayed with DAPI, LC3, and MERGE, respectively. Relative fluorescence intensity of LC3 (e) was compared between groups. ^∗^ *P* < 0.05, ^∗∗^ *P* < 0.01, and ^∗∗∗^ *P* < 0.001.

As we identified the role of ATL-III in improving CKD-induced skeletal muscle atrophy in vivo, we next investigated the role of ATL-III in vitro. TNF-*α* was used to induce oxidative stress and inflammation models in C2C12 myoblasts. The effect of ATL-III on the viability of C2C12 myoblasts was measured. After incubation with different concentrations (20, 30, 50, and 100 *μ*M) of ATL-III for 24 h, we found that low concentrations (20-50 *μ*M) of ATL-III did not induce significant cytotoxicity, while high concentrations (100 *μ*M) of ATL-III was toxic to C2C12 myoblasts (Figure 4(a)). To find the most appropriate concentration of ATL-III for studies in vitro, the cell proliferation and apoptosis assays were performed. C2C12 myoblasts treated with TNF-*α* were incubated with ATL-III (20, 30, and 50 *μ*M) for 24 h. The result of the cell proliferation showed that the concentration of 50 *μ*M displayed a better effect of promoting cell proliferation than 20 *μ*M or 30 *μ*M (Figure 4(b)). Similarly, the antiapoptotic effect of ATL-III confirmed by Hoechst 33342 dye staining assay showed that TNF-*α*-treated cells exhibited apoptotic morphological characteristics with condensed DNA/fragmented nuclei and high intensity of blue staining due to enhanced membrane permeability and altered chromosome DNA structure in apoptotic cells. In contrast, the number of apoptotic cells induced by TNF-*α* was remarkably decreased after treatment with ATL-III, and also the antiapoptotic effect of ATL-III was dose-dependent (Figure 4(c)). So we choose the concentration of 50 *μ*M as the most appropriate concentration of ATL-III for in vitro studies. The immunofluorescence assay indicated that the abundance and intensity of LC3 and signs of autophagy were markedly increased upon TNF-*α* induction in C2C12 myoblasts (*P* < 0.001). However, after treatment with ATL-III (50 *μ*M) for 24 hours, the LC3 expression was significantly decreased (Figure 4(d)). Relative fluorescence intensity of LC3 was used to display differences between groups (Figure 4(e)). Overall, these results suggested that the TNF-*α*-induced apoptosis of C2C12 myoblasts was attenuated by ATL-III together with the inhibition of autophagy.

### 3.5. ATL-III Inhibited TNF-*α*-Induced ROS and Antioxidant Enzyme in C2C12 Myoblasts

**Figure 5:**
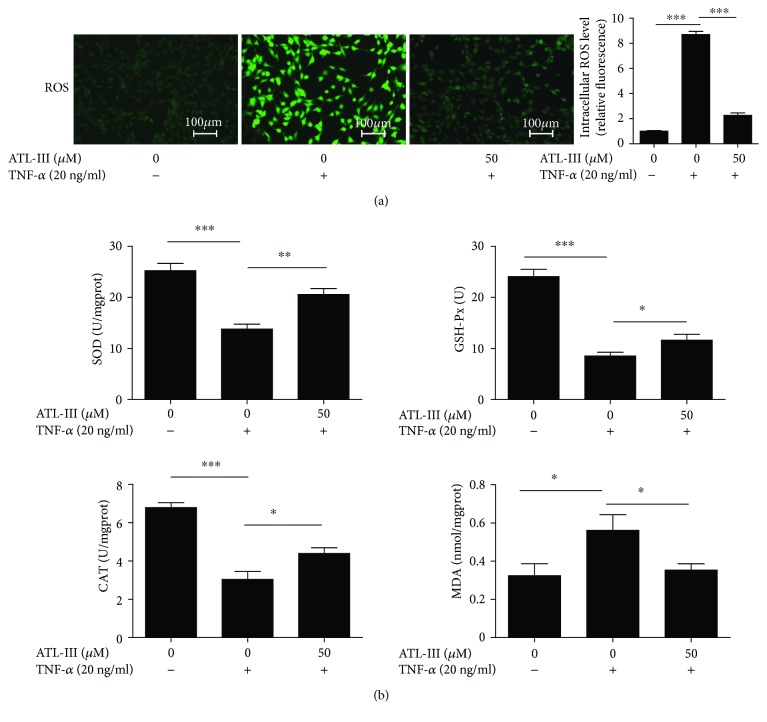
ATL-III inhibits TNF-*α*-induced oxidative stress in C2C12 myoblasts. C2C12 myoblasts were treated with ATL-III in the presence or absence of TNF-*α* (20 ng/ml) for 24 h and then loaded with H2DCF-DA for 30 min and observed under a fluorescence microscope; the quantification showed that the much higher level of ROS induced by TNF-*α* was decreased dramatically by ATL-III in C2C12 myoblasts (a). After trypsin digestion, the cells were centrifuged at room temperature for 1000 rpm/min for 10 minutes; then, the supernatant was removed, leaving cell sedimentation for detection. The activity of SOD, MDA, and GSH-Px and the level of CAT in cells are shown in the histograms (b). ^∗^ *P* < 0.05, ^∗∗^ *P* < 0.01, and ^∗∗∗^ *P* < 0.001.

We analyzed the oxidative stress-related factors, including ROS, MDA, SOD, and GSH-Px in C2C12 myoblasts. Our results show that TNF-*α* treatment led to strong ROS fluorescence, whereas ATL-III treatment decreased the staining intensity (Figure 5(a)). TNF-*α* also induced decreased SOD, CAT, and GSH-Px activity and increased MDA content, which were reversed by ATL-III treatment (Figure 5(b)). These results indicate that ATL-III has a protective effect against TNF-*α*-induced ROS production and can be used as a ROS scavenger.

### 3.6. ATL-III Decreased Cell Autophagy via the Oxidative Stress-Mediated PI3K/AKT/mTOR Pathway

**Figure 6:**
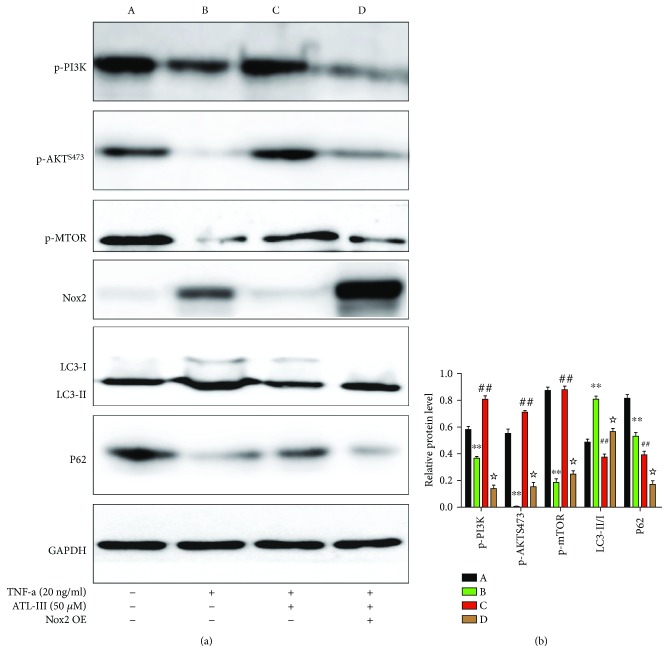
ATL-III ameliorated TNF-*α*-induced apoptosis in C2C12 myoblasts through the PI3K/AKT/mTOR pathway. C2C12 myoblasts were incubated with ATL-III in the presence or absence of TNF-*α* (20 ng/ml) for 24 h. After treatment and protein quantification, the expression levels of p-PI3K, p-AKT (Ser473), p-mTOR, LC3, and P62 were tested by western blotting. GAPDH was used as a loading control. The signaling pathway was explored again after overexpression of Nox2 (a). The densitometric analysis of images is shown in (b). ^∗∗^ *P* < 0.01, all compared with the A group; ^##^ *P* < 0.01, all compared with the B group; ^☆^ *P* < 0.01, all compared with the B group.

We examined whether the beneficial effect of ATL-III on muscle wasting was related to the oxidative stress-mediated PI3K/AKT/mTOR signaling pathway in C2C12 myoblasts. Western blotting showed that the treatment of ATL-III could increase the expression of p-PI3K, p-AKT (Ser473), and p-mTOR, which was decreased in C2C12 myoblasts induced by TNF-*α*. Furthermore, suppression of the PI3K/AKT/mTOR signaling pathway caused increased autophagy, as indicated by a higher expression of LC3-II and a lower expression of p62 when oxidative stress was increased by TNF-*α* treatment. These results indicated that ATL-III inhibited oxidative stress and autophagy probably by downregulating the expression of PI3K/AKT/mTOR. In addition, whether the expression change of p-PI3K, p-AKT, and p-mTOR influenced by ATL-III would be altered by ROS production needed to be observed. As Nox2 acts as the main source of ROS in skeletal muscle, we overexpressed Nox2 to increase ROS production. As we expected, when ROS was overproduced by the overexpression of Nox2, the therapeutic effect of ATL-III was compromised and the expression of p-PI3K, p-AKT, and p-mTOR was also downregulated. And also the reduced expression of LC3-II and the increased expression of P62 were both reversed when Nox2 was overexpressed. All the results showed that ATL-III played a protective role in CKD through the oxidative stress-mediated PI3K/AKT/mTOR pathway (Figure 6).

## 4. Discussion

Mitochondrial dysfunction and decreased levels of antioxidant enzymes lead to increased ROS production, which commonly causes oxidative stress and contributes to CKD-induced muscle wasting. In this study, we tested the hypothesis that ATL-III has a beneficial impact on improving muscle wasting during CKD by reducing the production of ROS and alleviating oxidative stress. We demonstrated that ATL-III could prevent CKD-induced muscle atrophy and reduce the levels of inflammatory factors (TNF-*α*, CRP, IL-1*β*, and IL-6) in muscles and serum in CKD rats, and ATL-III also displayed its effect on promoting the transition from M1 (proinflammatory) to M2 (anti-inflammatory) phenotype in macrophages in muscles of CKD rats. Furthermore, mitochondrial morphology was also restored and the levels of antioxidant enzymes such as SOD, GSH-Px, and CAT increased after treatment with ATL-III in CKD model rats. Thus, the production of ROS was reduced in skeletal muscle of ATL-III-treated rats. In addition, the results reported in this study provided evidence of the direct and/or indirect effects of oxidative stress and autophagy in muscle wasting of CKD, as autophagy can be significantly alleviated via the oxidative stress-mediated PI3K/AKT/mTOR pathway. We identified this potential mechanism through the overexpression of Nox2, which is considered to be the main source of ROS in pathological conditions in muscles.

ATL-III, known as the major component of Atractylodes rhizome, seems to have anti-inflammatory and antitumor functions, regulates gastrointestinal function, and promotes the absorption of nutrients according to Chinese pharmacopeia. The previous studies demonstrated that ATL-III significantly prevented cell death and cell membrane damage in a dose-dependent manner [20] and inhibited the production of proinflammatory cytokines, such as IL-6, IL-1*β*, TNF-*α*, and IL-8 [21], which was similar with our results in CKD model rats. In addition, ATL-III administration was beneficial for skeletal muscle wasting by reducing oxidative stress and autophagy through inhibition of the generation of ROS. As further evidence, ATL-III also alleviated the level of MDA, one of the products of lipid peroxidation.

Reactive oxygen species (ROS) are a single-electron reduction product of oxygen in the body. ROS leak from the respiratory chain and consume approximately 2% of oxygen before it is delivered to the terminal oxidase [22]. Mitochondria are an important source of ROS, which are produced by normal metabolism in the body [23]. Mitochondrial dysfunction was observed in skeletal muscle of CKD rats. We believed that the mitochondrial dysfunction was not only a consequence of the CKD condition but also played a critical role in CKD-induced skeletal muscle atrophy. On one hand, the recent studies showed that CKD condition promoted the accumulation of indoxyl sulfate (IS), a uremic toxin, which accelerated mitochondrial dysfunction via the induction of oxidative stress in skeletal muscle cells [24, 25]. On the other hand, impaired mitochondrial function, reduced muscle mitochondrial mass, and decreased energy production in skeletal muscle, and also the oxidative injury to mitochondrial components set up a vicious cycle of increased ROS generation, leading to exacerbation skeletal muscle dystrophy [26, 27]. ROS act as the second messengers in cell signaling and play a beneficial role when maintained at low or moderate concentrations [28]. Under physiological conditions, a large amount of ROS is produced through the respiratory burst mechanism when phagocytic cells are stimulated by the cell membrane [29]. These ROS act as the main medium to exert phagocytosis and killing effects [30]. However, under pathological conditions, due to loss of the normal balance between ROS generation and elimination, pathological reactions are often induced in cells and tissues [31]. Oxidative stress is the imbalance between oxidation and antioxidation, with a trend toward an increasingly oxidative environment [32, 33]. Oxidative stress leads to inflammatory infiltration of neutrophils and increased secretion of proteases, resulting in more oxidative intermediates [34]. As a negative effect induced by free radicals in the body that is considered a key factor in aging and disease [35], oxidative stress is often manifested by increased production of ROS and injury to many cellular organelles, proteins [36], lipids, and membranes [37], which affects muscle function [38]. Under oxidative stress, antioxidant defense systems can remove reactive species through enzymatic antioxidants such as SOD, GSH-Px, and CAT and nonenzymatic antioxidants such as antioxidant vitamins, trace elements, coenzymes, and cofactors [39]. MDA is formed as an end-product of lipid peroxidation [40] and can serve as an indicator of the latter.

The mitochondrial respiratory chain enzyme complex on the mitochondrial inner membrane is the main site for the production of ROS [41] and the target organ for ROS damage. Nicotinamide adenine dinucleotide phosphate oxidase (NOX) is a membrane protein widely distributed in tissues and organs of the body [42]. Because it can reduce oxygen molecules to superoxide anions through NADPH-dependent single-electron reduction, it is the main source of ROS in vivo and is also the only mechanism of direct ROS production in vivo. NADPH oxidases have emerged as the main (or initial) source of ROS in skeletal muscle cells [43]. Skeletal muscles express three isoforms of NADPH oxidases (Nox1, Nox2, and Nox4) that have been identified as critical modulators of redox homeostasis [44]. Nox2 acts as the main source of skeletal muscle ROS during contractions, participates in insulin signaling and glucose transport, and mediates the myocyte response to osmotic stress [45].

The dual roles of ROS in muscle likely act as a “double-edged sword” because ROS act as signaling molecules that regulate mitochondrial energy metabolism, induce the expression of antioxidant enzymes, and regulate the formation of additional ROS by feedback in exercise, but mitochondrial oxidative stress contributes to skeletal muscle dysfunction in chronic diseases due to oxidative damage [46].

Several studies have suggested that autophagy is activated by oxidative stress. ROS can initiate autophagosome formation and autophagic degradation by acting as cellular signaling molecules [12, 47–49]. However, an increasing number of studies have reported that autophagy plays a role in aggravating skeletal muscle injury in the last stage of CKD [6, 50, 51]. Oxidative stress is involved in autophagy-associated remodeling of the mitochondrial network by the removal of dysfunctional mitochondria from atrophic chronic obstructive pulmonary disease (COPD) muscle cells.

Although the mechanisms involved in the regulation of autophagy by ROS during skeletal muscle wasting are not yet known, studies have suggested that several signaling pathways participate in this regulation. ROS have been suggested to be able to induce autophagy by regulating the activation of the PI3K/AKT/mTOR signaling pathway [52, 53]. A model of muscle atrophy by disuse demonstrated that ROS can inhibit AKT/mTOR signaling and consequently induce autophagy [54]. However, a skeletal muscle model using dystrophic mdx mice revealed that Nox2-derived ROS can activate the Src/PI3K/AKT pathway and subsequently mTOR, leading to autophagy inhibition. Existing evidence has revealed a mechanism by which Nox2-specific oxidative stress impairs autophagy through Src kinase-dependent activation of the PI3K/AKT/mTOR pathway.

Our results also showed that ATL-III administration could increase the body weight and wet weight of the gastrocnemius (Gastroc), soleus muscle, and tibial anterior (TA) muscles in CKD rats, and this benefit was demonstrated by reducing oxidative stress and autophagy through inhibition of the generation of ROS. Furthermore, autophagy not only played a beneficial role in removing damaged mitochondria but also aggravated skeletal muscle atrophy in model CKD rats. ATL-III inhibited autophagy and improved skeletal muscle wasting by reducing the generation of ROS produced by Nox2, thereby increasing the level of PI3K/AKT/mTOR and enhancing the inhibition of autophagy in vitro. Therefore, our study indicated that ATL-III might serve as a potential protective drug against muscle wasting through the activation of the oxidative stress-mediated PI3K/AKT/mTOR pathway; however, the precise molecular mechanism needs further research to be done.

## 5. Conclusions

First, we studied the effect of ATL-III administration by gavage in SD rats and compared the results of SCr, BUN, and urinary protein levels, skeletal muscle mass, and the inflammatory state among different groups. Second, mitochondrial morphology under TEM and the activity of SOD, GSH-Px, CAT, and MDA were studied to clarify the role of oxidative stress in skeletal muscle atrophy. APs, ALs, and the expression of LC3-II and p62 were assessed to detect the effect of ATL-III on autophagy. To shed further light on the mechanism, the protein levels of p-PI3K, p-AKT, p-mTOR, LC3-II, and p62 were tested by Western blot in TNF-*α*-induced C2C12 myoblasts and skeletal muscles in CKD rats, and reverse validation was performed by the overexpression of Nox2. In conclusion, we provide evidence that ATL-III can ameliorate skeletal muscle wasting induced by CKD by alleviating oxidative stress and autophagy through the oxidative stress-mediated PI3K/AKT/mTOR pathway.

## Figures and Tables

**Table 1:** Biochemical data evaluating kidney function.

	Sham	Model	ATL-III
BUN (mmol/l)	5.86 ± 0.29	12.17±1.18^∗∗∗^	9.95 ± 1.21^#^
Serum creatinine (mmol/l)	29.50 ± 2.43	64.83±6.55^∗∗∗^	47.67 ± 2.34^###^
Serum albumin (g/l)	49.56 ± 5.01	32.42±4.06^∗∗∗^	38.27 ± 2.17^###^
Urinary protein (mg/24 h)	9.41 ± 1.42	32.16±7.13^∗∗∗^	13.95 ± 1.81^###^

^∗∗∗^ *P* < 0.001 compared with the sham group, ^#^ *P* < 0.05 compared with the model group, and ^###^ *P* < 0.001 compared with the model group.

**Table 2:** Body weight and muscle mass in different groups.

	Control	Model	ATL-III
Body weight (g)	542.72 ± 39.99	406.15±60.94^∗∗^	438.92 ± 18.77^#^
Gastroc MWW (mg)	3290.4 ± 75.5	1165.8±74.3^∗∗∗^	2838.6 ± 63.5^###^
Sol MWW (mg)	632.8 ± 22.6	413.7±23.4^∗∗∗^	527.4 ± 29.6^###^
TA MWW (mg)	1120.2 ± 53.4	657.9±64.1^∗∗∗^	863.8 ± 62.2^###^

^∗∗^ *P* < 0.01 compared with the sham group, ^∗∗∗^ *P* < 0.001 compared with the sham group, ^#^ *P* < 0.05 compared with the model group, and ^###^ *P* < 0.001 compared with the model group.

## Data Availability

All the data supporting these results are shown in the paper and are available from the corresponding authors.

## Supplementary Materials

Supplementary Materials The supplementary materials include 2 figures. Supplementary Figure 1: macrophage marker immunofluorescence in skeletal muscles within the sham group, CKD group, and ATL-III treatment group. Supplementary Figure 2: effects of autophagy inhibitor 3-MA on skeletal muscle atrophy and autophagy in muscles in CKD rats.
