# Supplementary material for: Atractylenolide III Attenuates Muscle Wasting in Chronic Kidney Disease via the Oxidative Stress-Mediated PI3K/AKT/mTOR Pathway
Source: Oxid Med Cell Longev. 2019 Apr 18;2019:1875471. doi: 10.1155/2019/1875471 (PMC6501186; doi:10.1155/2019/1875471)
Supplement: Supplementary Materials — The supplementary materials include 2 figures. Supplementary Figure 1: macrophage marker immunofluorescence in skeletal muscles within the sham group, CKD group, and ATL-III treatment group. Supplementary Figure 2: effects of autophagy inhibitor 3-MA on skeletal muscle atrophy and autophagy in muscles in CKD rats. [file 1875471.f1.pdf]

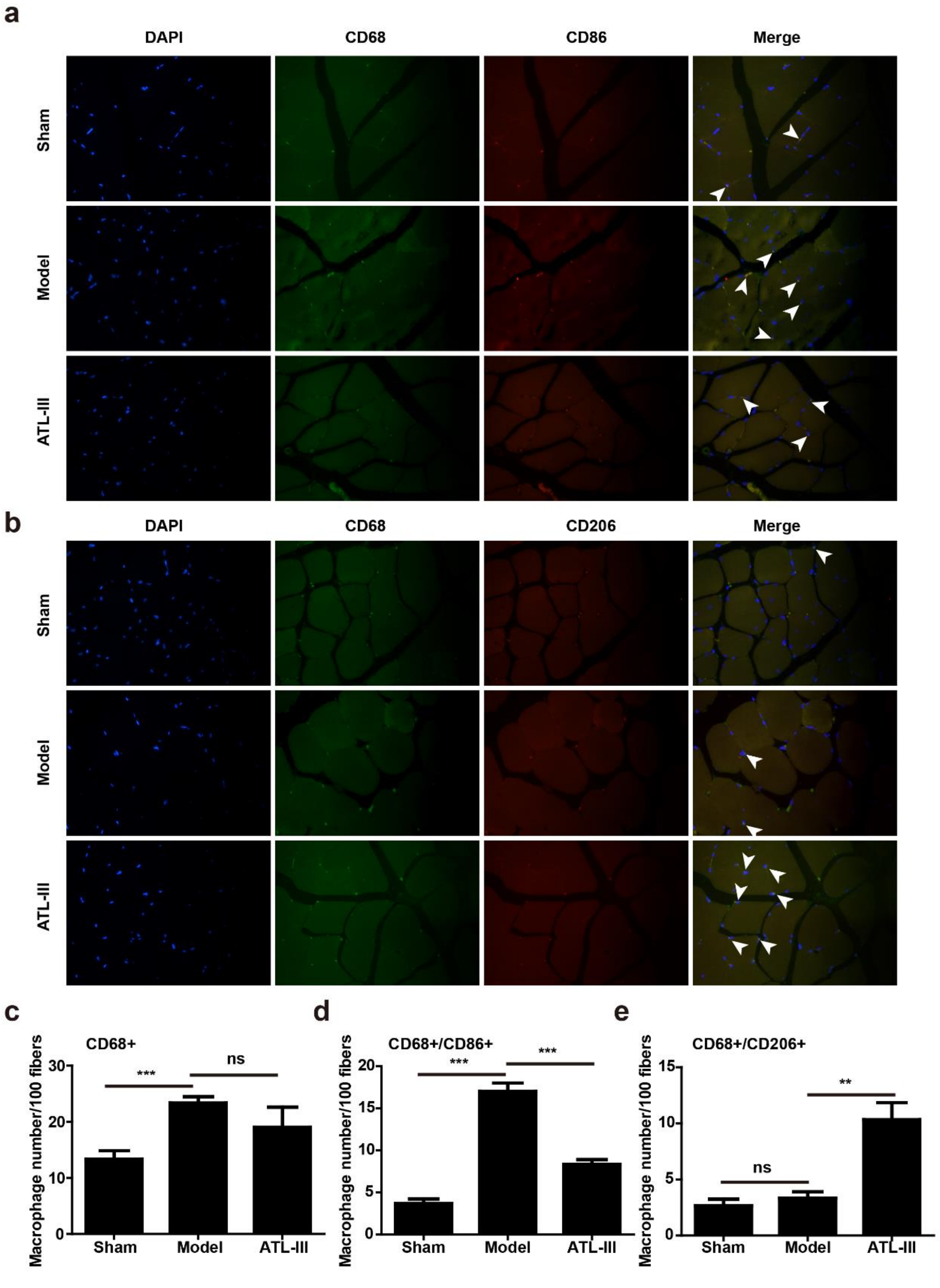

FIGURE S1. Macrophage markers immunofluorescence in skeletal muscles within the sham group, CKD group and ATL-III treatment group.

(a-b) Representative immunofluorescence images demonstrating CD68+ macrophages, CD68+ / CD86+ macrophages (M1), and CD68+ /CD206+ macrophages (M2) in

muscle biopsies of sham group, CKD group and ATL-III treatment group. (c-e) Quantification of CD68+ macrophages, CD68+ / CD86+ macrophages (M1), and CD68+ /CD206+ macrophages (M2) in muscle biopsies of sham group, CKD group and ATL-III treatment group. *p < 0.05, **p < 0.01. ***p < 0.001, ns: no significance.

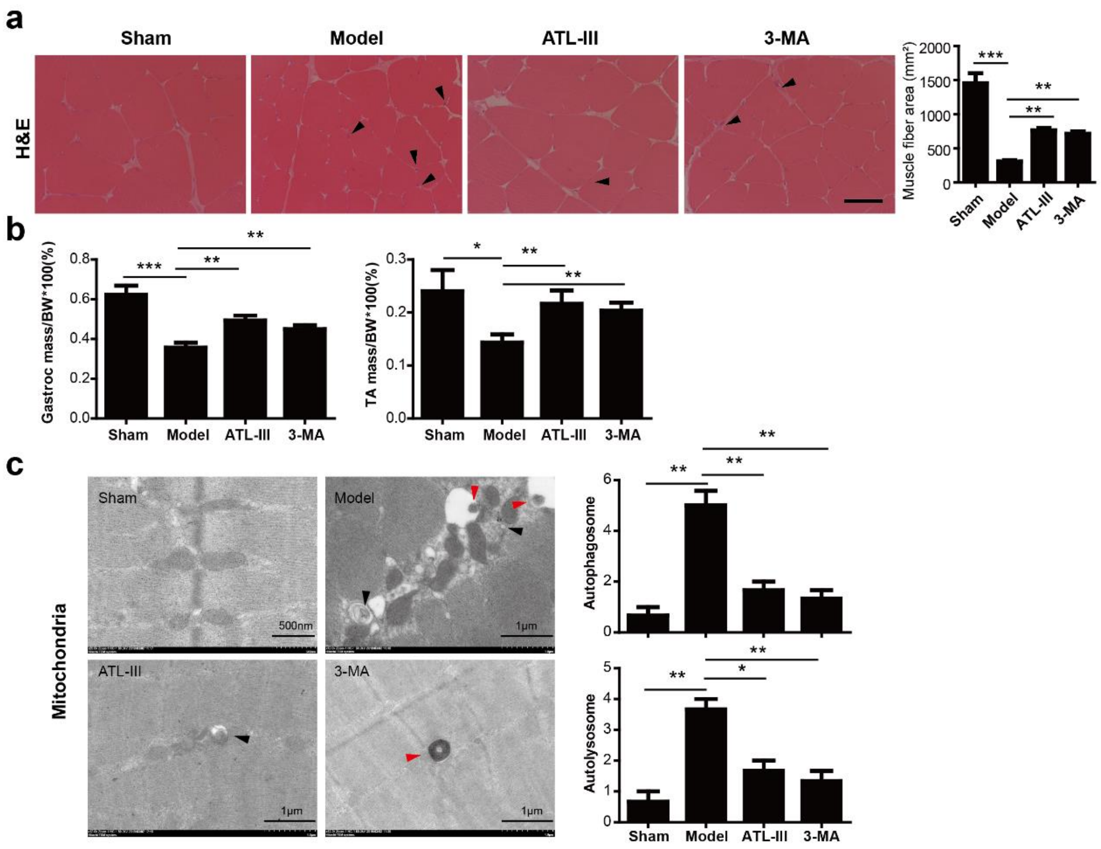

FIGURE S2. Effects of autophagy inhibitors 3-MA on skeletal muscle atrophy and autophagy in muscles in CKD rats.

(a) showed the representative images of tissue sections from sham, model, ATL-III and 3-MA groups. The black arrow indicates atrophic muscle fibers. The average muscle fiber area (μm2) of different groups was compared by one-way ANOVA. (b) Quantify and demonstrate the ratio of Gastroc and TA muscle mass normalized to total body weight. (c) The number of autophagosomes and autolysosomes in muscles of CKD models could be reduced significantly by ATL-III and 3-MA. Representative TEM images showing autophagosome structures (denoted by black triangles) and autolysosome structures (denoted by red triangles). *p < 0.05, **p < 0.01. ***p < 0.001.
